# Epilepsy and Ecstatic Experiences: The Role of the Insula

**DOI:** 10.3390/brainsci11111384

**Published:** 2021-10-22

**Authors:** Fabienne Picard, Peter Bossaerts, Fabrice Bartolomei

**Affiliations:** 1 Department of Clinical Neurosciences, University Hospitals and Medical School of Geneva, 1211 Geneva, Switzerland; 2 Department of Finance, University of Melbourne, Parkville 3010, Australia; peter.bossaerts@unimelb.edu.au; 3 Faculty of Economics, University of Cambridge, Cambridge CB3 9DD, UK; 4 Clinical Neurophysiology and Epileptology Department, Timone Hospitals, 13360 Marseille, France; Fabrice.BARTOLOMEI@ap-hm.fr

**Keywords:** ecstatic epilepsy, ecstatic aura, anterior insula, mental clarity, certainty, predictive coding, active inference, interoception

## Abstract

Ecstatic epilepsy is a rare form of focal epilepsy in which the aura (beginning of the seizures) consists of a blissful state of mental clarity/feeling of certainty. Such a state has also been described as a “religious” or mystical experience. While this form of epilepsy has long been recognized as a temporal lobe epilepsy, we have accumulated evidence converging toward the location of the symptomatogenic zone in the dorsal anterior insula during the 10 last years. The neurocognitive hypothesis for the genesis of a mental clarity is the suppression of the interoceptive prediction errors and of the unexpected surprise associated with any incoming internal or external signal, usually processed by the dorsal anterior insula. This mimics a perfect prediction of the world and induces a feeling of certainty. The ecstatic epilepsy is thus an amazing model for the role of anterior insula in uncertainty and surprise.

## 1. Introduction

Epilepsies are brain diseases of various etiology characterized by abnormal electric activities in brain networks. Epilepsy has always provided a unique opportunity to get a better understanding of cortical functions and, more particularly, of complex cognitive functions. In particular, the use of intracranial electrode investigations (recording and stimulation) for presurgical purpose has led to a better knowledge of the pathological phenomena but also of the cerebral functioning. For instance, a large variety of studies about the epileptic symptoms of “déjà vu” and of “reminiscence of memory”, including the “Proust phenomenon”, have helped to understand the different roles of the mesial temporal lobe regions in memory [1,2,3,4]. Ecstatic epilepsy is another example of a phenomenon of epileptic origin which helps to disentangle the neural correlates of specific cognitive states [5,6]. The ecstatic epileptic aura mimics experiences which have been described for centuries and have fascinated philosophers, psychologists, and theologians, among other people. These experiences sometimes are referred to, and described as, “mystical experiences”. Thus, ecstatic epilepsy brings a tremendous opportunity to study the neuronal correlates of this specific state of consciousness.

As a reminder, an epileptic seizure is a dynamic process. In focal epilepsies (as opposed to generalized epilepsies), the epileptic discharge starts in one region of one hemisphere and may progressively spread across the brain, with visible evolution of the clinical symptoms within a few seconds to a few minutes. An epileptic aura may inaugurate the seizure, in the form of symptoms which are only experienced by the patient and are not discernible to the people around. After the aura, the seizure can evolve into a loss of contact and, in some cases, into convulsions.

Ecstatic epilepsy is a rare form of focal epilepsy in which the aura consists of a blissful state, experienced as a vivid feeling of lucidity and plenitude. The first detailed description of ecstatic seizures dates from Dostoevsky in the 19th century [7]. He described them in his letters to friends and used the descriptions of his own seizures for characters of his novels, such as Prince Mychkine in the novel *The Idiot*. Around 50 cases of patients with ecstatic epilepsy have been described in the literature, mostly as reports of single cases [8]. However, this form of focal epilepsy is very likely under-estimated because people are often reluctant to speak about the ecstatic symptoms. They find it difficult to find the right words to express what they experienced, and they fear being taken for mentally deranged.

## 2. Similarities of the Ecstatic Aura with the Mystical Experience

A thorough interview of all the epileptic patients treated at the University Hospitals of Geneva and the University Hospital of Marseille, led to a precise description of the symptoms felt during the focal seizures. The interviews led to identification of about 20 cases with ecstatic epilepsy within the last 10 years. Clear common features of ecstatic auras emerged: a sense of physical well-being, a feeling of heightened self-awareness (constant) and heightened vividness of the environment (inconstant) with descriptions of a sensation of mental clarity, sometimes called enlightenment, ultimate reality, revelation, knowledge, or certainty. This sensation of mental clarity was the main finding, reported in all cases. Some patients reported, additionally, a sense of unity with the environment. The experience was associated with an intense positive affect in nearly all patients, even if some feared that the ecstatic experience would possibly evolve into a convulsive (tonic–clonic) seizure after having lived it.

- *The American psychophysiologist and philosopher William James had a great interest in similar experiences that he called “religious” or which are called “mystical” [9]. For him, the genuine “mystical experience” provides a pointer to a reality that is more likely true than the one we usually live (“insight into depths of truth”). According to him, a mystical experience is defined by four features:*
- *Ineffability in the way “the experience defies expression”*
- *A noetic quality which is a state of knowledge, or “insight into depths of truth”, unaffected by the discursive intellect; thus mystical states are illuminations, revelations, full of significance and importance*
- *Transiency*
- *Passivity*

## 3. Arguments for the Role of the Insula in the Ecstatic Experience

Looking to identify a brain region that could be the source of this range of symptoms, namely, physical well-being, heightened self-awareness, and intense positive affect, around 12 years ago we explored the anterior insula [5]. According to Craig [10], within the insula there is a posterior-to -anterior processing of interoceptive signals, coming from the inside the body and contextualized by external signals. Interoceptive signals are processed with additional cognitive information arriving to the insula, and result, in the anterior insula, in a representation in the form of subjective feelings. One neuroimaging study was particularly convincing in demonstrating this gradient within the insula, from a posterior physical representation of an (external) signal of cold on the hand to an anterior emotional integration [11]: the activity (blood flow) in the posterior insula correlated with the objective intensity of the cold temperature, while the activity in the anterior insula correlated with the subjective appreciation (feeling) of the temperature intensity.

**Figure 1:**
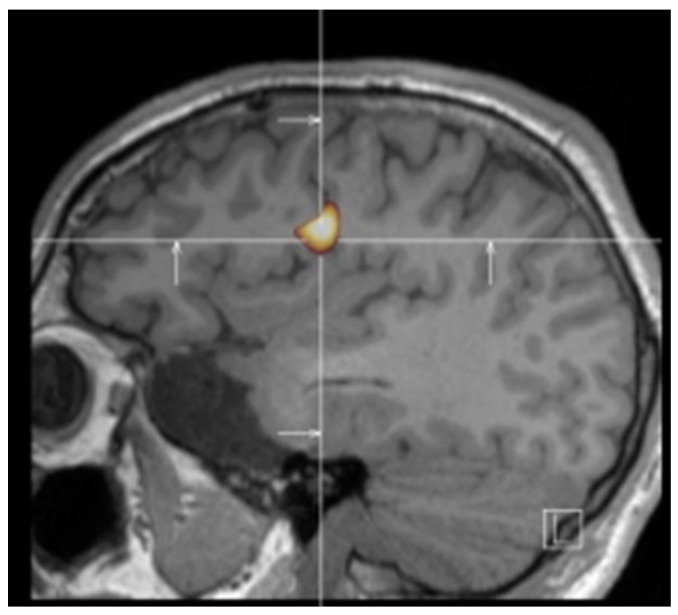
Cerebral MRI (sagittal view) and ictal/interictal technetium-99m HMPAO (99mTc-HMPAO) SPECT subtraction using BRASS analysis program in a patient with ecstatic epilepsy. It shows an increased blood flow maximal at the junction of the right dorsal mid-insula and central operculum. Adapted from a Figure published in Cortex 49 (2013), F. Picard, State of belief, subjective certainty, and bliss as a product of cortical dysfunction, page 2495, Reproduced with permission from Elsevier.

Support for the hypothesis that the anterior insula plays a crucial role in the ecstatic symptoms came through nuclear imaging studies in a few patients with ecstatic epilepsy. In one patient who already had a surgery with a temporal lobe resection, a single-photon-emission computed tomography (SPECT) using technetium-99m ethyl cysteinate dimer (99mTc-ECD) during an ecstatic seizure showed an increased blood flow in the anterior insula [5]. In a second patient, an increased blood flow also appeared in the dorsal part of the anterior/mid-insula, when performing the subtraction between a SPECT during a seizure and a SPECT outside a seizure (Figure 1) [6]. Even more convincing evidence in favor of anterior insula as the source of the ecstatic experience came in a study of a patient from Marseille in France [12]. She had an ecstatic epilepsy which was drug-resistant. After surface EEG evaluation, she had a pre-surgical evaluation of her epilepsy, with intracerebral electrodes (stereo-electroencephalography, SEEG) implanted within the right temporal lobe and insular cortex. A spontaneous recorded seizure involved first the right mesiotemporal region and then showed a propagation in less than 1 s in the dorsal anterior insula, concomitant with the ecstatic symptoms. Importantly, the stimulation of the electrode within the dorsal part of the anterior insula at 50 Hz during 3 s induced the ecstatic aura in a reproducible way. There was no post-stimulation discharge, supporting the fact that the symptoms were related to that insular region only. Stimulation of the mesiotemporal region or of other regions did not induce the ecstatic aura.

**Figure 2:**
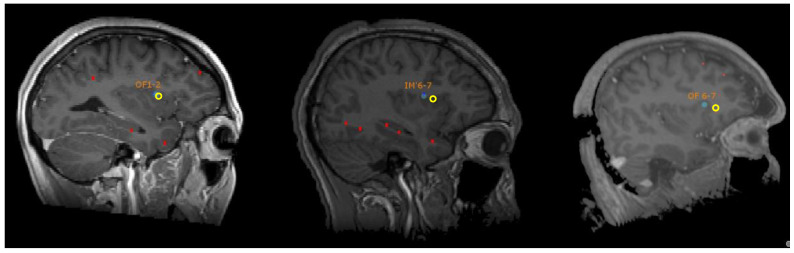
Localization of the contacts inducing an ecstatic aura in the operculo-insular region in three patients, in yellow. Adapted from a Figure published in Brain Stimulation 12(5) (2019), F. Bartolomei, S. Lagarde, D. Scavarda, R. Carron, C.G. Bénar, and F. Picard, The role of the dorsal anterior insula in ecstatic sensation revealed by direct electrical brain stimulation, page 1123, Reproduced with permission from Elsevier.

**Figure 3:**
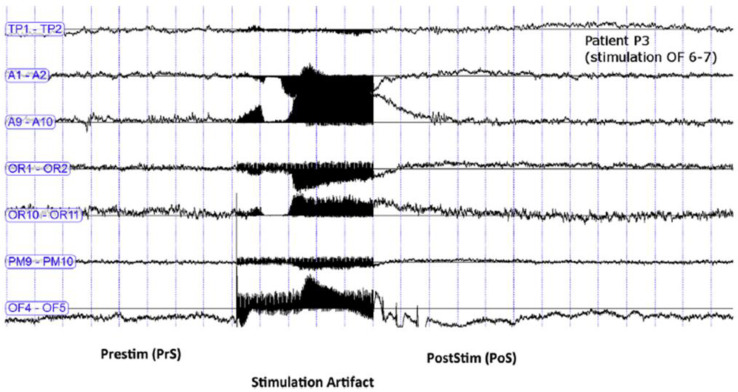
Stereo-electroencephalography (SEEG) recording during a stimulation inducing an ecstatic aura, in Patient 3, showing the detail of seven bipolar channels. The figure shows the SEEG signals and the artifact of electrical stimulation applied in the insula (50 Hz, 1 ms pulse width, 1.6 mA) for 5 s. There is no visible after-discharge. Each line represents a bipolar lead representative of a brain region: TP1–2: temporal pole; A1–2: amygdala; A9–10: middle temporal gyrus; 0R1–2: orbitofrontal cortex; OR 10–11: dorsolateral prefrontal cortex; PM9–10: premotor cortex; and OF4–5: anterior insula. Adapted from a Figure published in Brain Stimulation 12(5) (2019), F. Bartolomei, S. Lagarde, D. Scavarda, R. Carron, C.G. Bénar, and F. Picard. The role of the dorsal anterior insula in ecstatic sensation revealed by direct electrical brain stimulation, page 1123, Reproduced with permission from Elsevier.

Subsequently, ecstatic auras could be reproduced in two other patients suffering also from an ecstatic epilepsy in Marseille, through the electrical stimulation of the dorsal part of the anterior insula (Figure 2) on the left side for one patient and on the right side for the other one (with 5-s stimulation at 50 Hz, pulse width 1 ms, see Figure 3) [13]. Functional connectivity analysis on SEEG signals pooled from the three cases was applied to study the interactions between brain regions during these stimulations. A measure of directed connectivity (out-degrees) showed that the insula was the leading structure in the period following the stimulations and coinciding with the emergence of ecstatic symptoms. According to personal communications, ecstatic auras have since been reproduced in several other patients through the stimulation of the insula.

Nevertheless, in all cases, patients had already suffered from ecstatic epilepsy. Therefore, one cannot exclude the possibility that insular network had been altered through repeated prior epileptic discharges, favoring appearance of the ecstatic phenomenon at the time of the stimulation of the dorsal anterior insula. Conclusive evidence for a crucial role for anterior insula did not emerge until recently, when we managed to induce ecstatic experience by electrical stimulation of the dorsal part of the anterior insula in a patient who suffered from a temporal lobe epilepsy with non-ecstatic auras (manuscript under review).

## 4. Neurocognitive Hypothesis

The study of the neurobiological foundations of decision-making has led to the development of the theory of predictive coding [14,15]. According to this theory, the brain functions as a predictive machine. This means that it acts primarily through predictions. It does not wait for stimuli from the outside world or the bodily reactions to those stimuli. The predictions are referred to as “top–down signals” [16]. A “reference model” generates the predictions. The model builds on past experience and may be partly innate [15]. When external or interoceptive stimuli actually do arrive, the resulting “bottom–up” signals arriving in the brain are compared to the top–down ones. Any mismatch produces a prediction error. If the magnitude of the prediction error is large, the reference model is updated, to make it more successful in future forecasts and, hence, to make future actions more effective. Thus, surprise, which corresponds to the magnitude of the prediction error causes revisions of the reference model, to ensure that it better reflects reality.

**Figure 4:**
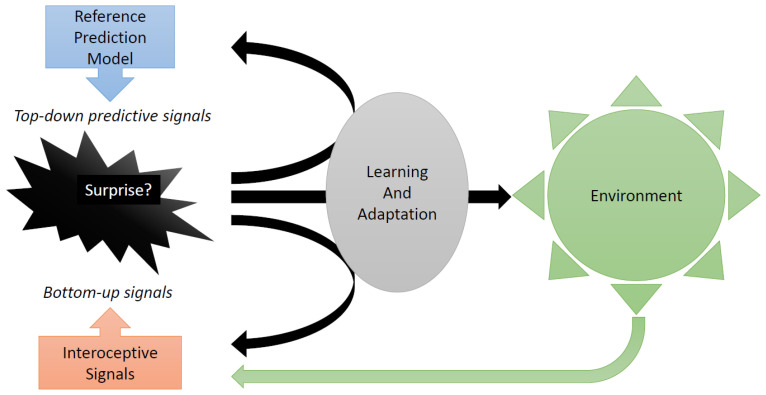
Proposed Insula Function (in black). Insula receives interoceptive bottom–up signals as well as top–down predictive signals. The latter emerge as the output of a “reference model” that forecasts interoceptive signals. Anterior insula compares the bottom–up and top–down signals and produces a surprise signal when the discrepancy is larger than expected. Upon surprise, the decision-maker is made aware of the need to learn and adapt. This involves updating the reference model (**black arrow pointing up**), actively interfering in the environment (**black arrow pointing to right**) and emotional preparation for future changes in the environment (**black arrow pointing down**). Some time later, the environment (**green**) produces new stimuli, which in turn affect interoceptive signals. The resulting bottom–up signals are again compared to (**top–down**) predictions from the updated reference model. If the size of prediction errors is as expected, there is no more surprise, and no more learning and adaptation.

In more recent versions of the theory of predictive coding [17,18,19], the opposite happens as well: in the case of surprise, the decision-maker (“agent”) attempts to bring reality in line with the reference model. The agent actively searches for environments that better reflect the reference model. In other words, the agent attempts to alter the environment so as to experience less surprise. The agent searches for better control of uncertainty. This “active inference” enriches learning paradigms. For indeed, there are two ways to enhance predictive coding: one is to improve the reference model; the second one is to interfere with the environment and to prepare emotionally for this interference, with the purpose of making the reference model predict better (see Figure 4).

Active inference makes novel implications. Most importantly, as long as surprise is acceptable, actions are maintained. When surprise increases, the need to adapt emerges. Learning and adaptation thus emerge only after excessive surprise. We believe that behavior is not guided by an urge to optimize, i.e., to become the best possible, even if, under certain circumstances, optimality can be generated [20]. This is in contrast to standard theories, such as in *Q Learning* on which machine learning is built [21]. In Q learning, prediction errors are used solely to improve the reference model, until it becomes optimal. That is, mistakes are used exclusively to update the reference model until it makes the best possible forecasts.

In opposition to Q learning, in real life, learning and adaptation stop when an acceptable level of uncertainty (not too high) is reached. This is consistent with active inference, where the agent does not optimize, but “satisfices” (to use a term originally suggested in [22]). A satisficing agent stops adapting when reaching a “bliss point,” which emerges when interaction with the environment generates uncertainty that is in line with the agent’s reference model. If the agent cannot act upon its environment in a way that generates the desired amount of uncertainty (i.e., not too high) and if the agent fails to draw the right conclusions—which is that the reference model has to be adapted—then frustration ensues. As such, active inference theories may shed new light on anxiety and depression disorders. Among others, people who exhibit less tolerance for uncertainty should be more susceptible to anxiety and depression [23].

As to interoception, i.e., the perception of the physiological state of the body, the comparison between predictions and real incoming signals has been shown to take place within the insula [24]. The insula also tracks the magnitude of these prediction errors, that is, the insula tracks the surprise. The interoceptive experience, as well as the resulting emotions, can be thought of as the result of the surprise signals rather than of the incoming interoceptive signals themselves [24,25].

Evidence has been mounting over the last two decades that the dorsal anterior insula plays a crucial role in tracking surprise and uncertainty, in the form of a signal that compares how far outcomes are from predictions. This role emerges in a variety of settings, from complex social situations [19,20] down to simple probabilistic [21,22] and perceptual tasks [23]. Besides these functional MRI studies linking surprise and the anterior insula, there are human pathologies which also support this association. E.g., anxiety proneness correlates with increased dorsal anterior insula activity [26]; intolerance to uncertainty is associated with increased anterior insular reactivity [27], while intolerance to uncertainty is more acute in anxiety disorders [28].

During an ecstatic seizure, we have demonstrated that there is an epileptic discharge within the anterior insula [12]. Our hypothesis as to the resulting ecstatic experience is based on the role of the anterior insula as comparator between predictions and outcomes, particularly in the field of interoception [6]. We here extend this hypothesis to electrical stimulation of the anterior insula. Electrical stimulation induces an increase in synchrony of neuronal firing. The increased synchrony could prevent anterior insula from reaching the minimal level of differentiation necessary to process the mismatch between interoceptive signals and their predictions and, hence, prevent the anterior insula from generating surprise. Consequently, the patient reaches a bliss point; s/he experiences an unusual and hard-to-articulate sensation, of being in control of, or of completely understanding, his/her environment.

## 5. Conclusions

The ecstatic epilepsy is being revealed here as an amazing model for the role of the anterior insula in building awareness of uncertainty and surprise. It has the potential to elucidate the role of surprise in driving adaptation and control, and ultimately the level of physical and mental well-being. Future work could exploit the ability to create ecstatic symptoms to decipher the causal effect of disruption of neural firing in the dorsal anterior insula on feelings and behavior.

There is still a need to understand why only a subset of subjects experienced an ecstatic phenomenon as the result of an epileptic discharge in, or of electrical stimulation of, the dorsal anterior insula. Potential explanations include differences in brain connectivity or differences in the autonomic nervous system, but one cannot exclude that discharge or stimulation may be insufficient to have an impact on feelings.

Induced ecstatic auras could provide a unique opportunity to understand the role of surprise in generating anxiety-related symptoms of some neuropsychiatric disorders. Ability to induce feelings of “bliss” through stimulation in patients suffering from depression may provide a path towards novel therapies, to temporarily alleviate symptoms, and, in the long run, to cure them.

## Data Availability

Data on the patients are available on request to the corresponding author.
